# The Insane in France
**Statistics of the Establishments for the Insane in France, from 1842 to 1853 inclusive*. By M. Legoyt.


**Published:** 1863-04

**Authors:** 


### Art. XIV.—THE INSANE IN FRANCE.*

(*From the American Journal of Insanity.*)

In 1843, the *Bureau de la Statistique Générale* published a series of tables, showing the condition and management of Insane Asylums in France, from 1835 to 1844 inclusive. In 1857,

---

* *Statistics of the Establishments for the Insane in France, from* 1842 *to* 1853 *inclusive.* By M. Legoyt.

a continuation of the same series was published, giving similar statistics for the twelve years from 1842 to 1853 inclusive, and bringing the report forward to the close of 1853.

The extent of this work and the laborious nature of its details seem astonishing to one not familiar with the minute precision and the ingenious exactness, which characterize the labours of the French statisticians. The published work is composed of two parts. The first part, relative to the eleven years 1842-52, gives the general results of progress from year to year; with tables of admissions, discharges, &c.; the number of patients for each department, and for each asylum in particular; its admissions, discharges, and deaths for each year. The second part is devoted specially to the statistics of the year 1853, and is even more minute and specific. Under the head of "Administrative Management," it details not only the admissions and discharges, but the number of lunatics to be found in every department of France, either in public or private charge; the price per diem paid for their maintenance and treatment; together with the relative share in these expenses borne by Government, by the various departments, by each commune, and even by the different hospitals and private families throughout the empire.

Under the head of "Medical and Physiological Observations," it gives as minutely as possible a series of facts, which enables the reader to appreciate the influence exerted upon the development of insanity, upon the mortality and the results of treatment in this unfortunate class, by sex, by age, by civil condition, by degree of education, by occupation, by season of the year, and by social position. It contains also some well-digested hints upon the probable causes of lunacy, making the usual triple distinction of the same into predisposing, physical, and moral.

Complete as these tables seem to be, and as they doubtless are in most respects, there are two sources of error which we must mention at the outset, and which, we regret to say, must necessarily affect the exactness of the results, and detract somewhat from their value in the minds of specialists. Of these the first is, the failure to classify separately the idiots and cretins which are found in the various asylums. The second is, that of counting among the admissions many cases which have been discharged apparently well, and shortly re-admitted after a relapse; also those who have made their escape from the asylums, and are soon retaken; and finally, those cases which are merely transferred from one asylum to another. To count all these cases as admissions is manifestly unjust. Both these errors will be corrected in subsequent reports, and official measures, it appears, have been taken since 1856, to secure a degree of exactness in recording the statistics, which will render them almost absolutely perfect.

At the close of the year 1855, the whole number of institutions in France devoted entirely or in part to the treatment of lunatics, was 111. Of these, 65 were public institutions, and 46 were private. Under public asylums may be reckoned those which pertain to the Government, to the various departments, to communes, and to almshouses, thus:—

| | | |
|---|---|---|
| Belonging to | Government (Maison de Charenton) | 1 |
| ,, | Departments | 37 |
| ,, | Communes | 1 |
| ,, | Almshouses | 26 |
| | Total | 65 |

In this number are included certain establishments which are not strictly public asylums—*i.e.*, not authorized as such; for example, certain poorhouses, where persons suffering from mental disease, chiefly idiots and incurables, are harboured or maintained by public sanction, if not at public expense. This, however, does not apply to those establishments where cases may be admitted temporarily, to await a transfer elsewhere.*

| | |
|---|---|
| French asylums appropriated exclusively to male patients | 11 |
| ,, ,, ,, female patients | 17 |
| ,, in which both sexes are received | 83 |

Of the 28 which are appropriated exclusively to either sex, are reckoned,—Departmental asylums, 8; Almshouse-asylums, 7; Private asylums, 13.

The 111 asylums (including those of every class), are distributed among 61 departments. The largest number in any one department (de la Seine) is 16. Several other departments have a number of asylums ranging from 2 to 8; 41 have each a single asylum within their limits, while in 25 departments no provision whatever has been made for the treatment of this unfortunate class.

This unequal distribution of so large a number of asylums,

---

* Previous to 1844, it is difficult to determine the exact number of asylums in France, either public or private. This uncertainty is due to the incompleteness of returns made by officials to Government, and the unreliable nature of many facts stated.

Six of the 37 departmental asylums were opened during the ten years, 1844-54, and two or three others have been changed into such from almshouse-asylums (*hospices*). During the same ten years the number of private asylums has but slightly varied. Three have been closed, either voluntarily or by public authority, and four others have been newly licensed.

In the number of almshouse establishments devoted to the reception of lunatics the official reports mention no instance of a new licence, while many have been changed into almshouse-asylums or have been suppressed altogether.

seems most unfortunate and unwise. The population of the 25 departments which are unprovided with even a single asylum was (according to the census of 1851), 8,908,923, or one-fourth of the entire population of the empire. It seems, therefore, that one-fourth of all the families of France, in case of the discovery of mental alienation in their midst, have no resource but to seek at the hands of a neighbouring department the care and the curative measures the emergency demands, and which unhappily their own limits do not furnish. This state of things diminishes the chances for recovery, as well as limits the number of those who would seek the benefits of an institution. Families are often, from a humane but unwise impulse, unwilling to be separated entirely from a relative under such circumstances, and thus great numbers of unfortunates remain at home, receiving no treatment and rarely recovering; a burden or a terror to their families, and too frequently even propagating their own sad disease without restraint.

To such destitution as this, fortunately, the United States presents no parallel. We may add, in regard to the geography of the twenty-five departments thus unprovided, that—

| | |
|---|---|
| 10 belong to the South of France | 3 belong to the East of France. |
| 9 „ Centre „ | 3 „ North „ |

All the western departments are provided with at least *one* asylum each.

In classifying the 111 asylums of France according to their capacity of accommodation, we use the tabular form, as follows:—

| Capacity of Asylums. | Asylums of State, departments, or communes. | Almshouse-asylums. | Private asylums. | Totals. |
|---|---|---|---|---|
| For less than 50 patients . . . . . . . . . . . | 1 | 6 | 25 | 32 |
| 50 to 100 „ . . . . . . . . . . | 2 | 4 | 5 | 11 |
| 100 to 200 „ . . . . . . . . . . | 10 | 6 | 9 | 25 |
| 200 to 300 „ . . . . . . . . . . | 10 | 3 | 1 | 14 |
| 300 to 400 „ . . . . . . . . . . | 8 | 2 | 2 | 12 |
| more than 400 . . . . . . . . . . . . | 8 | 5 | 4 | 17 |
| Totals . . . . . . . . . . . | 39 | 26 | 46 | 111 |

Of the 17 asylums which contain more than 400 patients, the first is the Salpêtrière, at Paris, which contained in 1853 the astonishing number of 1324 patients. This is an asylum exclusively for females. The total number of patients in these 17 asylums was, in 1853, 10,935, or more than 40 per cent. of all the lunatics under treatment in the whole of France.

POPULATION OF ALL THE ASYLUMS OF FRANCE SINCE 1835.

In 1835, the first census was taken of the insane under treatment throughout the empire. Since that time (excepting in 1850, when a diminution is observed, which is due to the ravages of the cholera in 1849), the number has increased year by year. We give an abstract of the tables, which will be sufficient for our purpose. The number of patients in all the French asylums was:—

| | | | |
|---|---|---|---|
| January 1, 1835 . . . | 10,539 | January 1, 1850 . . . | 20,061 |
| ,, 1840 . . . | 13,283 | ,, 1851 . . . | 21,353 |
| ,, 1845 . . . | 17,089 | ,, 1854 . . . | 24,524 |

Thus we see that this population doubled itself from 1835 to 1851, and its increase during 19 years was 13,985, or 133 per cent. In tracing this progressive advance from year to year, we are struck with the fact that the proportion of increase for several later years is lessened. This is most easily seen by arranging the 15 years, 1839-54, in periods of 5 years each, thus:—

| Period. | Increase of inmates. | Rate of increase for each period. | Ditto for each year. |
|---|---|---|---|
| 1839 to 1844 . . . . . . . . . | 3678 | 29·24 per cent. | 5·85 per cent. |
| 1844 to 1849 . . . . . . . . . | 3976 | 24·46 ,, | 4·89 ,, |
| 1849 to 1854 . . . . . . . . . | 4293 | 21·22 ,, | 4·24 ,, |

The annual rate of increase for the first period, 5·85 per cent., is reduced in the next 5 years to 4·39 per cent., and in the period, 1849-54, we find it 4·24 per cent.; a result which allows the hope that in a more or less distant future, the number of insane patients in France may cease to show any increase whatever.

The next tables in order in the French report are those relating to the classification of insane patients according to sex. This classification shows not only a preponderance of female cases under treatment, but this preponderance has of late years very considerably increased. From 1842 to 1854, the average number of patients under treatment was, males, 9,314; females, 10,177. In 1841 the proportion of females to males was as 102 to 100. From 1842 to 1854, it was as 109 to 100. The first impression from this might be, that females are more liable than males to mental disease; whereas we shall hereafter see that the reverse is the truth. The true explanation of the difference is found in the fact that male patients invariably remain a shorter time under treatment than females, and also that the deaths of males in asylums greatly exceed in number those of females.

PROPORTION OF LUNATICS UNDER TREATMENT TO THE WHOLE POPULATION OF FRANCE.

If we compare the number of patients in asylums during the years 1836, 1841, 1846, 1851, with the entire population of France, we find the following results :—

| Years. | Population of France. | Patients in asylums. | Proportion of patients to the population. |
|---|---|---|---|
| 1836 . . . | 33,540,910 | 11,091 | 33 to 100,000 or 1 to 3024 |
| 1841 . . . | 34,240,178 | 13,887 | 41 ,, ,, 2465 |
| 1846 . . . | 35,400,486 | 18,013 | 51 ,, ,, 1965 |
| 1851 . . . | 35,783,170 | 21,353 | 60 ,, ,, 1676 |

As regards sex, the proportions are as follows :—

| | | | |
|---|---|---|---|
| *Males* —In 1846 . . | 48 to 100,000 inhabitants, or 1 in | 2063 |
| 1851 . . | 57 ,, ,, ,, | 1731 |
| *Females*—In 1846 . . | 53 ,, ,, ,, | 1877 |
| 1851 . . | 61 ,, ,, ,, | 1625 |

It appears, therefore, that females under treatment exceed males by one-tenth. We have already attributed this preponderance of females to the constant excess of male discharges and deaths.

The next subject considered in the report, is that of—

LUNATICS MAINTAINED AT HOME, AND THEIR NUMBER IN PROPORTION TO THAT OF LUNATICS UNDER TREATMENT IN ASYLUMS.

According to the census of 1851, there were at that time in France 44,970 cases of mental disease. These are divided as follows :—

Under treatment in various establishments, 20,537, or 45 per cent.
At home . . . . . . . . . . . . . . 24,433, or 55 ,,

Total . . . . . . 44,970, or 1 in 796 of whole population

"The number here given of the cases in asylums," the Report adds, "may be somewhat under-estimated, since only those cases are reckoned which are actually under treatment, omitting all persons who may be temporarily placed in institutions, either for purposes of transfer or otherwise. The estimate, therefore, above given, is doubtless below the truth." With much more reason should we suspect the truth of the second estimate, that of the lunatics at home. The almost universal reluctance of friends to acknowledge cases of family derangement, and the desire, dictated by either humanity or policy, to conceal such facts from the authorities, to avoid the exposure of a public record, will necessarily operate to render such tables incomplete.

We may therefore be assured that the above estimate is at least not exaggerated, and allowing this fact, we come at once to the conclusion that 24,000 insane persons, or more than half of all the lunatics in France, are detained at home, and deprived of the benefits and the comforts of an asylum; not to mention the sacrifice of chances for recovery, which are universally admitted to attend a removal from the cares and associations and excitements of home. In view also of the public safety, if more than half a nation's lunatics be at large, or at least under the imperfect restraints of home, is society to have no assurance that these shall be harmless? In the constant liability to outbreak and disturbance, in the summing up of public and private anxiety, in the frequent recurrence of accidents and crime, which must unavoidably attend such a state of things, is there no added burden to the State, and no increased responsibility to the conservators of the public peace?

The details of these tables (for which we have no space) show that the six departments where are found most lunatics at home, belong to the twenty-five already alluded to as having no asylum whatever for insane cases; while the ten departments where are found the fewest lunatics at home, have at least one asylum each within their borders. This can surprise no one. The departments which are supplied with asylum-advantages, would naturally have fewer lunatics at home than the others. To make this still more plain, let us compare, in a tabular form, the number of the insane at home in the twenty-five departments, with the same class in the other sixty-one departments of France, thus:—

| Departments. | Population in 1851. | Insane at home in 1851. | Proportion in 100,000 inhabitants. |
|---|---|---|---|
| In the 25 departments having no asylum . . . . . . | 8,908,923 | 7,225 | 73·24 |
| In the 61 departments having one or more asylums . . | 26,874,227 | 17,208 | 64·03 |
| For the whole of France . | 35,783,170 | 24,433 | 68·28 |

Passing over Chapter III. of the Report, which relates to the details of the internal economy of the French asylums, we come to Chapter IV., which is devoted to the subject of "Admissions." Of this, the first subdivision is—

THE NUMBER AND INCREASE OF ADMISSIONS TO ASYLUMS SINCE 1853.

The constant increase already marked in the cases under treatment, is also noticed in the admissions.

| | | | | | |
|---|---|---|---|---|---|
| In 1835 | the number admitted to French asylums was | | | | 3917 |
| 1840 | „ | „ | „ | | 5433 |
| 1845 | „ | „ | „ | | 7518 |
| 1850 | „ | „ | „ | | 8184 |
| 1851 | „ | „ | „ | | 8592 |
| 1852 | „ | „ | „ | | 9782 |
| 1853 | „ | „ | „ | | 9081 |

During a period of nineteen years the whole number of admissions was 128,542, or an average of rather more than 6765 per annum. In 1852 the number reached its maximum. If we compare this with the number of admissions in 1835, we find it almost triple.

"What can be the cause" (we quote from the Report) "of this enormous augmentation? Is it a consequence of the increased number of our asylums, their enlargement, and their approved appliances for usefulness? Or, on the other hand, does insanity—as some have urged—claim annually, more and more victims? Can we believe that this cruel scourge follows the development of civilization itself? that it keeps pace with the progress of public education, with industrial and commercial activity, with the growth of public wealth, with the ardent strife for honour and for power, with the panting race for fortune, for ease and for luxury, which so peculiarly characterize the present age? The feverish excitements which are forced upon the mind by the uncertainties of political life, with its passions, its wranglings, its disappointments, and its snares—could these be regarded as fatal to reason? The revolutions, the financial and industrial crises, the unbridled speculations, the reverses of fortune, the ebb and flow of emigration, and those social convulsions which seem inevitable to the present constitution of society—would these necessarily produce a similar effect? These are grave questions, nor can we pretend to answer them until the sphere of our observation shall be enlarged, and especially until the census of many succeeding years shall allow us to compare the number of the insane at home with that of the insane treated in special asylums. For the present our investigations must remain incomplete."

There is, however, a partial answer given to the question, in the following considerations, which are wholly independent of psychological influences.

First, as we have already intimated, the erection in different parts of the country of new asylums, with increased accommodations for patients. Secondly, the great improvements in their management; the substitution of moral and rational means for the harsh treatment which once terrified families and disgraced our specialty; the attraction which is exerted more or less widely by the names of distinguished physicians, who are charged with the management of asylums; the gradual weakening of the opinion once so prevalent, that insanity is incurable; the moderate expense of maintaining patients in very many asylums; also, the moral and physical difficulties which beset the management and treatment of a lunatic at home; the greatly increased facilities for travelling, which allow of the easy conveyance of patients to almost any distance; and finally, and above all other considerations, the gratuitous treatment of the poor, in cases where reason is not irrecoverably lost. We may thus encourage the hope that if the number of those received annually in asylums be gradually augmented, on the other hand, the number of those maintained, or, we might almost say, *detained* at home, may in its turn diminish.

In addition to the other reasons for this recent increase of admissions, we must not omit to mention the numerous abuses which are practised by municipal authorities, and even by families, in imposing upon these establishments the charge of great numbers of paupers, under pretext of some form of mental alienation. These abuses have been made at different times the subject of special reports by the prefects of departments, and by the officers of asylums; but the full extent of the evil, it appears, has been only partially reached.

### THE INFLUENCE OF SOCIAL CRISES AND AGITATIONS OF THE PUBLIC MIND UPON THE DEVELOPMENT OF INSANITY.

We have already alluded to the opinions of certain authors, who assert that revolutions, wars, industrial crises, and generally all those unforeseen and startling events which from time to time interrupt the noiseless tenor of society, by the derangement or by the sacrifice of large interests, increase the number of admissions to asylums for the insane. But the statistics which we have examined do not confirm this observation. In fact, the year 1848, in spite of the revolution of February, and the extraordinary industrial disturbances which followed it, presents by comparison with 1847, fewer admissions. During the latter year they reached 7688, and in 1848 they fell to 7341, being 345 less. It is also worthy of note that this diminution was apparent even in the department of the Seine,* where the consequences of the revolution of 1848 were most severely felt. Of this department, the number of admissions to asylums, public and private, was:—

| | | | |
|---|---|---|---|
| In 1846 . . . . . . . | 1957 | In 1848 . . . . . . . | 1871 |
| 1847 . . . . . . . | 1876 | 1849 . . . . . . . | 1906 |

From these results one might almost be tempted to believe

* Containing the city of Paris and environs.

that great social crises have ceased to agitate materially the public, simply by the fact of their frequent occurrence.

We may, however, add, that the prolonged disturbance of the administrative service in 1848, under the influence of political prejudice, can explain in a great measure the diminution of admissions for that year. In 1852, when some of those most serious political events had been accomplished (but this time in the sense of a return to the establishment of order and authority), we observe the phenomenon of an unparalleled increase in the number of admissions (9782). This result, however, corresponds with the increase in the discharges before or after recovery. The discharges were :—

| | | | |
|---|---|---|---|
| In 1850 . . . . . . . . | 4402 | In 1852 [rising to the number] | 5442 |
| 1851 . . . . . . . . | 4519 | 1853 [falling again to] . . | 4872 |

The cholera of 1849 appears not to have exercised an influence corresponding to that of 1832, which according to the testimony of many observers, had produced a large number of cases of mental disease, as the result of panic. In 1848, the number of admissions to asylums was 7341, and in 1849 it was 7536; an increase only of 195.

PROPORTION WHICH THE ANNUAL ADMISSIONS TO ASYLUMS BEAR TO THE ENTIRE POPULATION OF FRANCE.

During the 19 years already selected for observation, the proportion for the whole of France was as follows:—

| | | | |
|---|---|---|---|
| From 1835 to 1838, | 1 admission to | 7661 | inhabitants. |
| 1839 to 1843 | ,, | 5649 | ,, |
| 1844 to 1848 | ,, | 4714 | ,, |
| 1849 to 1853 | ,, | 4144 | ,, |

For the single department of the Seine :—

| | | | |
|---|---|---|---|
| From 1835 to 1838, | 1 admission to | 595 | inhabitants. |
| 1839 to 1843 | ,, | 580 | ,, |
| 1844 to 1848 | ,, | 568 | ,, |
| 1849 to 1853 | ,, | 516 | ,, |

We thus find the proportion during the period 1849-53, for the single department of the Seine, more than ten times as great as that for all the other departments together. This is not surprising, when we consider that this department includes the city of Paris and its environs. The peculiar circumstances of a vast city like the capital of France, render it of course necessary to regard as dangerous, and to place in confinement, not only actual maniacs, but idiots and imbeciles, cases of senile dementia, and indeed every individual deprived of the power of self-contro To this cause may be added the just reputation which the metropolitan asylums enjoy throughout the empire, and also the advantages which they offer to wealthy families for placing their friends under treatment secretly, and thus avoiding the exposure of their occasional or hereditary infirmities.

ADMISSIONS ACCORDING TO SEX, FROM 1842 TO 1853.

The relative liability of the sexes to mental disease, is a subject upon which authors have widely differed. Some have regarded this infirmity as more usual among males, while others have insisted that it is more frequent among females, whose organization is more delicate and more impressible, the emotional faculties predominating in a more marked degree over those of the stronger sex. The following table seems to settle this question:—

| Period. | Males. | Females. | Total. | Per cent. of Males. | Per cent. Females. | Ratio of Males to Females. |
|---|---|---|---|---|---|---|
| Total number of admissions to Asylums in France, from 1842 to 1853 . . | 50,194 | 43,975 | 94,169 | 53·30 | 46·70 | 1·14 |

From the details of the table (of which the above is merely an abstract, giving the totals) it appears that during the twelve years, 1842-54, the number of annual male admissions has constantly exceeded that of females in a ratio which amounts to more than 14 per cent., and, what is remarkable, this average ratio, based upon the statistics of more than 94,000 admissions, varies only from 8 to 14 per cent. during the twelve years selected. And as in the entire population of France, there are more females than males, we may conclude with confidence that insanity is a disease to which men are more subject than women.

The same excess of male admissions over female, is recorded in the statistics of the public and private asylums of the department of the Seine:—

| Period. | Males. | Females. | Total. | Per cent. of Males. | Per cent. Females. | Ratio of Males to Females. |
|---|---|---|---|---|---|---|
| For the 12 years, 1842–54, total admissions . . . | 11,842 | 11,363 | 23,205 | 51·03 | 48·97 | 1·04 |

According to the census of 1851, and the annual average of admissions during the period of five years, 1849-54, we find for the whole of France—

1 admission to every 3864 of male population.
„ „ 4473 female „

In the department of the Seine—

1 admission to every 702 males | 1 admission to every 692 females.

INFLUENCE OF AGE.

The admissions to the various French asylums during the year 1853, are thus classified according to age:—

| Age. | No. of Admissions. | | |
|---|---|---|---|
| | Males. | Females. | Total. |
| Under 14 years . . . . | 310 | 256 | 566 |
| From 14 to 20 years . . . | 662 | 581 | 1243 |
| ,, 20 to 25 ,, . . . | 1274 | 1169 | 2443 |
| ,, 25 to 30 ,, . . . | 1850 | 1619 | 3469 |
| ,, 30 to 35 ,, . . . | 2312 | 1845 | 4157 |
| ,, 35 to 40 ,, . . . | 2272 | 2041 | 4313 |
| ,, 40 to 50 ,, . . . | 3447 | 3564 | 7011 |
| ,, 50 to 60 ,, . . . | 2035 | 2575 | 4610 |
| ,, 60 to 70 ,, . . . | 762 | 1260 | 2022 |
| 70 and over . . . . . . . | 303 | 536 | 839 |
| Age unknown . . . . . | 1186 | 1017 | 2203 |
| Totals . . . . | 16,413 | 16,463 | 32,876 |

If it were possible to regard the age at the time of admission as the date also of the attack, we should infer from this document that mental derangement does not often exhibit itself until after puberty. Commencing from that epoch, the disease moves on, so to speak, in a parallel line with the progress of the advancing intellect, and thus becomes more and more frequent until 35 or 40 years of age, the period of life when the intellectual development usually attains its maximum. Of 1000 cases of insanity, 141 declare themselves between these two periods. The danger of an acute attack diminishes gradually until old age, when we observe more of mental decrepitude, known as senile dementia.

Insanity in all its forms shows itself somewhat later in women than in men. Of 1000 male patients, the disease appeared in 570 before the fortieth year, while of 1000 females, 485 only became insane before that age. Between 50 and 60, females are much more liable to mental disturbance than males, the proportion being, in every 1000 cases, 167 females to 134 males. This result accords with the generally received opinion, that the critical period of woman's life predisposes to insanity.

To sum up all the cases under treatment in 1853, and take the average age at which they were admitted to treatment, we find it—

| | | | |
|---|---|---|---|
| For males . . . . . . . . . . . . . . | 39 years | 1 month | |
| females . . . . . . . . . . . . . | 41 ,, | 9 ,, | |
| both sexes . . . . . . . . . . . . | 40 ,, | 5 ,, | |

The mean age, therefore, of admission to asylums is forty years and five months.

INFLUENCE OF DOMESTIC RELATIONS.

The 32,876 insane persons under treatment in 1853 may be classified according to their domestic relations at the time of admission to the various French asylums, as follows:—

| Condition. | No. of Patients in 1853. | | |
|---|---|---|---|
| | Males. | Females. | Both sexes. |
| Single . . . . . . . . . . . . | 9278 | 8800 | 18,078 |
| Married. . . . . . . . . . . . | 4047 | 4446 | 8,493 |
| Widows or widowers . . . . . | 791 | 1888 | 2,679 |
| Unknown . . . . . . . . . . . | 2297 | 1329 | 3,626 |
| Totals . . . . . . . . | 16,413 | 16,463 | 32,876 |

We give also, classified as above, the entire population of France, deduction being made of those under fifteen years,* as follows:—

| Condition. | No. of Inhabitants of 15 years and upwards. | | |
|---|---|---|---|
| | Males. | Females. | Both sexes. |
| Single . . . . . . . . . | 5,010,616 | 4,549,944 | 9,560,560 |
| Married . . . . . . . | 6,986,223 | 6,948,828 | 13,935,051 |
| Widows or widowers . . | 836,509 | 1,687,583 | 2,524,092 |
| Totals . . . . | 12,833,348 | 13,186,355 | 26,019,703 |

One of the most remarkable facts brought to light by the above tables, is the large proportion of unmarried persons admitted to the asylums, being for both sexes 61·80 per cent. of all those under treatment.

In the whole population at large, deducting all under fifteen years, we find only 36·74 per cent. unmarried inhabitants, being a proportion of more than one-third less than we find in the asylums.

The same fact has already been noticed by certain psychologists, who have attributed it to a special predisposition to insanity on the part of those living in celibacy. "The solitude of the unmarried state," they say, "the absence of the cares, the affections, and the joys of the family, leaves a man unarmed

* As insanity is rarely noticed before the age of fifteen years, it would manifestly be unjust to establish a comparison between the unmarried, married and widowed, of the whole population, without excepting all whose youth will hardly allow of their being married.

This table is based upon the census of 1851.

against many of the trials and the temptations of married life. The hour of adversity arrives, he is plunged in sad thoughts, he encourages a sort of selfish melancholy, and yields to its influence. His reason is thus more exposed to wander than that of the married man, who in similar trials enjoys the sympathies of wife and children, and in the consciousness of his duties and responsibilities as a husband and father, he possesses a courage, resignation, and endurance, to which the bachelor is a stranger."

This opinion is doubtless well founded, but in explaining the difference in the admissions of this class, it should not be forgotten that, in the nature of the case, from the very isolation of the single man, his removal to an asylum might be a necessity, while a married man could receive oftentimes, in the bosom of his family, that care which might conduce to his recovery.

In 1857, the proportion which unmarried patients over fifteen years of age bore to the whole population at large, was—

| | |
|---|---|
| Of bachelors or maids . . . . . . . . . . | 1 in 529 inhabitants. |
| Of widows or widowers . . . . . . . . | 1 in 942 ,, |
| Of those married . . . . . . . . . . . | 1 in 1641 ,, |

PROFESSIONS AND VOCATIONS.

Of 32,876 inmates of the various French asylums in 1853, it was found impossible to ascertain the professions of more than 27,620. These are classified as follows:—

| Professions. | No. of Patients. | | | Whole No. of each profession (census of 1851). | Proportion of insane in each profession (in 1853) to population at large. |
|---|---|---|---|---|---|
| | Males. | Females. | Both sexes. | | |
| Liberal professions . . | 1970 | 1075 | 3,045 | 1,712,082 | 1 to 562 |
| Soldiers and sailors . | 718 | ... | 718 | 360,185 | 1 to 502 |
| Commercial pursuits . | 709 | 430 | 1,139 | 2,672,467 | 1 to 2347 |
| Mechanical ditto . . | 6418 | 4138 | 10,556 | 15,788,038 | 1 to 1495 |
| Servants, day-labourers, &c. . . . . . . . | 1791 | 2568 | 4,359 | 2,808,917 | 1 to 644 |
| Miscellaneous and no occupation . . . | 2553 | 5250 | 7,803 | 12,441,481 | 1 to 1594 |
| Totals . . . . | 14,159 | 13,461 | 27,620 | 35,783,170 | 1 to 1294 |

We must observe, in the first place, that one of these classes—viz., "soldiers and sailors," cannot strictly be compared with the others, since the war and navy departments provide for the immediate admission to some asylum of every soldier and sailor, without exception, affected with mental disease, while very many of the other classes are not thus provided for, and are never placed under treatment. It is therefore not surprising that the military population should offer, as is indicated above, an exceptional number of admissions to the various asylums.

Next to the "soldiers and sailors," the "liberal professions" furnish the largest proportion of insane cases. After these, in order, come "servants, domestics, day-labourers, &c.," then "mechanics," and finally, those engaged in "commercial pursuits."

The class of "liberal professions," according to the French Report, is made to include, in addition to the three ordinary departments of law, theology, and medicine, certain miscellaneous avocations, which will perhaps seem extraordinary to the American reader, and which will require a separate table for the explanation of these statistics:—

| Liberal Professions. | Population in 1851. | No. of cases treated. | Proportion of cases treated to whole population. |
|---|---|---|---|
| Artists (painters, sculptors, architects, engravers, musicians) . . . . . | 23,839 | 229 | 1 to 104 |
| Jurists (judges, advocates, notaries, lawyers, and bailiffs) . . . . . | 30,050 | 253 | 1 to 119 |
| Ecclesiastics (monks and nuns) . . . | 82,371 | 341 | 1 to 253 |
| Physicians (surgeons, apothecaries, and midwives) . . . . . . . . . . | 39,424 | 152 | 1 to 259 |
| Professors and men of letters . . . | 93,032 | 332 | 1 to 280 |
| Public office-holders and employés . . | 272,440 | 375 | 1 to 727 |
| Proprietors and tenants . . . . . | 1,170,926 | 1,363 | 1 to 806 |

From this table, we learn that the proportion of "artists" in the French asylums is nearly eight times greater than that of "proprietors and tenants," seven times that of "jurists," five times that of "priests and physicians," and four times that of "literary men."

Of the sum of all these classes, we find the large proportion of 1 insane person to every 205 inhabitants, while for the whole population, we have already noted the proportion as 1 to 1294 inhabitants.

This result confirms the generally-admitted view, that those pursuits which demand a continued exercise of the intellectual and reflective faculties, are most favourable to the development of mental disease.

In the category of "commercial pursuits," are reckoned all those who sell, either in a large or a small way, those products which are or are not the result of their own labour. Manufacturers, therefore, as well as merchants and those engaged in the various branches of commerce, must be included in the number.

In proportion to the number of individuals which it embraces, this class reckons a very small number of insane cases. In the asylums they stand as 1 to 25, while among the population at large, this class represents one-seventh of all the inhabitants of France.

Under the head of "mechanical pursuits," comprising the farmers and the artisans (strictly so called), we find that the proportion of insane cases under treatment by no means equals the proportion which this class bears to the whole population. This proportion reaches (making allowance here as elsewhere, in all statistics of population, for children and women living upon the wages of husbands) somewhat less than 44 per cent., and the proportion of their insane 38 per cent. The farming class furnished 3789 cases, and the industrial, although employing four or five times less individuals, furnished 6767, or nearly one-half more. Hence it appears that, in an equal population, the number of labourers and artisans admitted to insane asylums, exceeds by a large proportion that of the farmers. This fact agrees with the strongly marked predominance of insane cases which belongs to the population of large cities.

In the class of "servants, day-labourers, &c.," may be reckoned domestics attached to the farm, person, or house, and all who are employed for wages in whatever capacity—*e.g.*, cooks, coachmen, housekeepers, waiters, &c. If our documents are correct, the proportion of insane cases belonging to this category exceeds the general average by one-half. This can only be explained by the great number of single persons in this class of those devoted to the indoor service of families, and we have already seen that a majority of all the inmates of asylums are unmarried.

The sixth and last class, entitled "miscellaneous, and having no occupation," includes beggars, feeble persons, courtezans, children, and in short, all persons without any known means of livelihood, or whose occupation is not susceptible of classification. Nearly three-tenths of the insane in France (or 283 in 1000) belong to this category.

DEGREE OF EDUCATION OF INSANE CASES.

The classification of 32,876 cases treated in French asylums during 1853, according to the amount of education received previously to their admission, is shown as follows:—

| Degree of Education. | No. of patients (both sexes). | Ratio to 100 cases. |
|---|---|---|
| Able to read . . . . . . . . . . . | 3,795 | 11·54 |
| Able to read and write . . . . . . . . | 6,447 | 19·61 |
| More advanced acquirements . . . . . | 2,694 | 8·20 |
| No instruction, or unknown . . . . . | 19,940 | 60·65 |
| Totals . . . . . . . . . | 32,876 | 100·00 |

We have no educational statistics of the whole population with which to compare these figures, but the table shows that one-fifth of all the patients could read and write at the time of their admission; one-tenth could read only; one-twelfth had enjoyed the benefits of a superior education, while more than one-half are set down as having no education whatever, or the facts in their case were unknown.

PROBABLE CAUSES OF INSANITY IN THE CASES ADMITTED IN 1853.

The report makes the ordinary triple distinction of causes—viz., predisposing, physical and moral.

An abstract of 19,938 cases, arranged according to sex and probable causes, is found in the following table:—

| Causes. | Males. | Females. | Both sexes. | Proportion in 1000. |
|---|---|---|---|---|
| Predisposing causes . . . | 1410 | 1473 | 2883 | 144 |
| Physical causes . . . . | 5478 | 4286 | 9764 | 490 |
| Moral causes . . . . . | 3314 | 3977 | 7291 | 366 |
| Totals . . . . . | 10,202 | 9736 | 19,938 | 1000 |

We are doubtless safe in regarding the chief predisposing cause of insanity as hereditary predisposition, and from the above table we may conclude, therefore, that this cause is somewhat more active in the case of females than males. The predominance of physical over moral causes, as given in the table, should be taken with some allowance. It should not be forgotten that physical causes are always more palpable to the eye, and more easily detected, and an apparent physical cause may be sometimes accepted on a hasty observation as the true one, when a further inquiry might demonstrate its fallacy.

Moral causes appear to be more active in the case of females, and physical causes among males. This view, which the table furnishes, seems in the nature of the case to be a just one, and is moreover confirmed by the experience of all specialists. Of 9764 cases of insanity attributed to physical causes, the report assigns 2594, or 22 per cent., as the direct consequence of epilepsy or convulsions; 1502, or 15 per cent., as due to drunkenness, and 923, or 9 per cent., as due to hardship and privation.

The other physical causes are arranged as follows, in the decreasing order of their influence:—

| | | | |
|---|---|---|---|
| Effects of age (senile dementia) | 723 | Cerebral disturbances . . . | 146 |
| Onanism . . . . . . . . | 572 | Diseases of skin . . . . . . | 126 |
| Venereal excess . . . . . | 296 | Syphilis . . . . . . . . | 119 |
| Fevers . . . . . . . . . . | 283 | Excessive labour . . . . . . | 115 |
| Cerebral congestion . . . . | 257 | Slow and interrupted development among young girls . . | 79 |
| Suppression of menses . . . | 140 | Hydrocephalus . . . . . | 69 |
| Following childbirth . . . | 140 | Gestation and lactation* . . | 56 |
| Blows and injuries . . . . | 149 | | |

Of the moral causes of insanity, the Report gives as the most frequent and active, "chagrin following loss of property." 899 cases are set down as due to this cause, or 12 in every 100. After this are reckoned, in decreasing order, the following:—

| | | | |
|---|---|---|---|
| Religious excitement . . . . . | 894 | Excessive intellectual labour . . | 156 |
| Love . . . . . . . . . . . | 792 | Ordinary imprisonment . . . . | 54 |
| Violent emotions (shocks, &c.) . . | 698 | Nostalgia . . . . . . . . . | 48 |
| Pride . . . . . . . . . . . | 600 | Isolation and loneliness . . . . | 41 |
| Loss of friends . . . . . . . | 510 | Change from active life to one of leisure, or *vice versâ* . . . . | 32 |
| Disappointed ambition . . . . | 495 | Association with the insane . . . | 16 |
| Jealousy . . . . . . . . . . | 442 | Solitary imprisonment . . . . | 4 |
| Political events . . . . . . . | 308 | | |

From these tables it would appear that, setting aside hereditary predisposition, the most active causes of mental derangement are epilepsy, convulsions, and drunkenness. After these may be reckoned hardships of all kinds, pecuniary loss, and religious excitement, and finally love, old age, violent passions, pride, and onanism. These various causes together constitute nearly one-half the recognised causes of insanity.

ADMISSIONS ACCORDING TO THE SEASON OF THE YEAR.

Are not attacks of mental disease more frequent during some months of the year than others? It is scarcely possible to answer this question with certainty or with any scientific accuracy; since, although the attack may occasionally be sudden and well-marked, it is more commonly slow in its approach and its progress, and is oftentimes preceded by symptoms which only a professional observer can detect.

Some light, however, is thrown upon the question by the

---

* In this table it is somewhat difficult to determine the purpose in separating the ill effects of "gestation and lactation" from the other accidents "following childbirth." The same may also be said of "cerebral congestions" and "cerebral disturbances."

following table. Of 27,413 patients admitted to asylums in 1853, it shows, taking each sex separately, in what month each individual was admitted, and the proportion of the monthly admissions (each month being reckoned at 31 days), to the total number of admissions, 12,000:—

| Months. | No. of Admissions. | | | Proportion in 12,000 admissions. |
|---|---|---|---|---|
| | Males. | Females. | Both sexes. | |
| January . . . . . | 984 | 965 | 1949 | 838 |
| February . . . . . | 850 | 988 | 1838 | 874 |
| March . . . . . . . | 977 | 1023 | 2000 | 859 |
| April . . . . . . . | 1075 | 1027 | 2102 | 933 |
| May . . . . . . . . | 1231 | 1168 | 2399 | 1031 |
| June . . . . . . . | 1247 | 1164 | 2411 | 1070 |
| July . . . . . . . | 1329 | 1383 | 2712 | 1166 |
| August . . . . . . . | 1169 | 1146 | 2315 | 995 |
| September . . . . . | 1195 | 1233 | 2428 | 1078 |
| October . . . . . . . | 1152 | 1269 | 2421 | 1040 |
| November . . . . . | 1396 | 1167 | 3563 | 1138 |
| December . . . . . | 1155 | 1120 | 2275 | 978 |
| Total . . . . | 13,760 | 13,653 | 27,413 | 12,000 |

Classified according to the four climacteric seasons of the year, we have:—

| Season. | No. of Admissions. | | | Proportion in 12,000 admissions. |
|---|---|---|---|---|
| | Males. | Females. | Both sexes. | |
| Winter (Dec. Jan. Feb.) . | 2989 | 3073 | 6062 | 2690 |
| Spring (Mar. Apr. May) . | 3283 | 3218 | 6501 | 2823 |
| Summer (June, July, Aug.) | 3745 | 3693 | 7438 | 3231 |
| Autumn (Sep. Oct. Nov.) . | 3743 | 3699 | 7412 | 3256 |
| Totals . . . . | 13,760 | 13,653 | 27,413 | 12,000 |

It thus appears that the maximum of admissions occurs in summer and autumn, and that the minimum of admissions belongs to the winter season.

Counting both sexes together, we find that the maximum of admissions is in July; the same is also true of the female sex alone. For males, the maximum is in November. This difference, which has not been noticed hitherto by specialists, requires to be confirmed by future observations.

The minimum of admissions also does not fall in the same month for each sex. For males, the minimum is in February, and for females, January. Of the two added together, the minimum falls in January.

DURATION OF THE DISEASE PREVIOUS TO ADMISSION IN ASYLUMS.

Of the 32,876 insane cases under treatment in 1853, only 14,693 present any data from which we may infer the duration of the disease previous to their admission. Of these the statistics are as follows:—

| Duration to time of admission. | Both sexes. |
|---|---|
| 1 month or less | 1297 |
| 1 month to 6 | 2569 |
| 6 months to 1 year | 1594 |
| 1 year to 2 years | 1375 |
| 2 years and over | 2446 |
| Since birth | 1888 |
| Indefinite period, short | 1132 |
| ,, long | 2392 |
| Total | 14,693 |

To make this table more exact, we should first deduct the 1888 cases whose disease dates from birth. This will leave 12,805. If for greater convenience this number be represented by 1000, we find the period of derangement previous to being placed in an asylum, was—

Less than a month for 101 cases.
1 to 6 months ,, 200 ,,
6 to 12 months ,, 125 ,,
1 to 2 years for 108 cases.
More than 2 years for 466 cases.

If these statistics be correct, then nearly one-half of all the unfortunates whom we find under treatment in the asylums, are not placed there until more than two years after the invasion of the disease. No wonder, therefore, need be excited by the large number of incurables which encumber our institutions.

COMPLICATIONS AND AGGRAVATING CIRCUMSTANCES.

Insanity is often complicated with paralysis and epilepsy, and this more frequently in the case of males than females. This will be inferred from the following table, which gives for each sex separately, 1st. The number of cases treated in 1853, in which these fearful disorders were noticed as complications; 2nd. The relative per centage of the two sexes thus affected; 3rd. The proportion of epileptics and paralytics in 1000 cases under treatment:—

| Sex. | Cases of Complication. | | Per cent. of each. | | Proportion of each in 1000 cases. | |
|---|---|---|---|---|---|---|
| | Paralysis. | Epilepsy. | Paralysis. | Epilepsy. | Paralysis. | Epilepsy. |
| Males | 986 | 1462 | 65·99 | 57·85 | 60 | 88 |
| Females | 508 | 1065 | 34·01 | 42·15 | 31 | 64 |
| Both sexes | 1494 | 2527 | 100·00 | 100·00 | 45 | 77 |

It will be observed that the liability of males to paralysis is nearly twice as great as that of females; the cases being as 60 to 31. The liability of males to epilepsy also is greater than that of females, although less strongly marked, being as 88 to 64.

### NUMBER OF RELAPSED CASES.

Among the 32,876 cases which form the basis of our statistics, we find 1635 noted as cases of relapse. This is in the proportion of 49 to 1000 cases under treatment. Of these relapses, 831 were males, and 804 were females, or in the proportion of 50 to 48 cases in 100. That a relapse is a more frequent occurrence among males is naturally explained by the greater predisposition of that sex to insanity, which has been already demonstrated. On the other hand, it is well known that in the cases of males, who, as husbands or fathers, are indispensable to the support of their families, physicians having them in charge are humanely prompted to shorten as much as possible the term of their separation from home. Thus, oftentimes, their period of convalescence is unwisely curtailed, rendering the liability to relapse of course much greater. This circumstance is worthy of consideration in an estimate of this nature. Of 1395 admissions to the hospitals of Bicêtre and Salpêtrière during the year 1853, 221 were cases of relapse, or in the proportion of 15·84 per cent. Of these 221 relapses, there were—

| | |
|---|---|
| 127 for the 1st time. | 5 for the 5th time. |
| 53 ,, 2nd ,, | 4 ,, 6th ,, |
| 18 ,, 3rd ,, | 1 ,, 10th ,, |
| 9 ,, 4th ,, | 1 even for the 14th. |

In more than one-half the cases, the relapse occurs during the first year of their recovery.

### PLACE OF RESIDENCE OF CASES UNDER TREATMENT IN 1853.

If we consider as "city population" that of every commune numbering at least 2000 inhabitants, and as "rural population" that of all the other communes, we find that the cases treated in 1853 may be divided according to their residence at the time of admission, as follows:—

| | |
|---|---|
| Living in towns | 12,972 |
| ,, country | 14,536 |
| Residence unknown | 5,368 |
| Total | 32,876 |

For the whole population of France, the census of 1851 gives, according to a similar classification—

| | |
|---|---|
| Living in cities and towns | 8,951,525, or 25½ per cent. |
| ,, country | 26,166,114, or 74½ ,, |
| Total | 35,117,639 |

The proportion between the city and rural population being as 1 to 3, the rural districts ought to furnish thrice as many patients as the cities. Now of 1000 cases admitted in 1853, 472 were from cities and towns, and 528 from the rural districts; from which we may infer, that the patients of city origin are more numerous than those from the country. Several specialists have already noted this fact, and they have generally agreed that among a crowded population, and especially in large cities, the development of luxury, the cultivation of the passions, the agitations, excesses, and various disorders of society, industrial crises with their attendant misfortunes and miseries, &c., are in the highest degree favourable to mental alienation.

We do not know to what extent this opinion, notwithstanding its apparent plausibility, is well founded, but it is not impossible that the predominance of lunatics in cities over the rural districts, may be attributed less to moral and emotional causes than to the restrictions which society is compelled, in a crowded population, to impose upon that unfortunate class. Considerations of public order and safety require that, in cities, all persons deprived of their reason, whatever may be their age and the nature of their affection, should be regarded as dangerous, and therefore placed in confinement by official authority.*

On the other hand, in the country, where lunatics are generally well known, where they are easily watched, and where their acts cannot, therefore, have the same dangerous consequences to the public peace, the administration leaves to the care of their respective families, those who show themselves to be harmless. Hence we may infer, that if lunatics of rural origin are relatively less numerous among the patients treated in asylums, they would doubtless be found much more numerous among the class of those maintained at home. This is confirmed by the figures of the following table, compiled from the census of 1851 (Vol. XIV. of *Statistique de France*) :—

| Residence. | Population in 1851. | No. of Lunatics maintained at home. | Proportion of this class to population. |
|---|---|---|---|
| In 363 chief towns of arrondissements | 6,406,557 | 1,856 | 1 to 3452 |
| In other towns and communes . . . | 29,376,613 | 22,577 | 1 to 1301 |
| Totals . . . . . . . | 35,783,170 | 24,433 | 1 to 1464 |

* "Each prefect is, in the circumscription of his department, the only person charged by and responsible to the police; and consequently he is the only judge of the propriety there may be of allowing an insane person to go at large, or of the necessity of confining him." (Ministerial decisions of Nov. 13, 1846; extract from letter addressed to *M. le Prefet de Seine-et-Marne.*)

In this table, the classification of lunatics does not exactly correspond with that of lunatics treated in asylums. Yet it cannot be misunderstood that the predominance of the urban element among the cases admitted to asylums, corresponds with the predominance of the rural element among those cases which are maintained at home. Thus the two facts may be regarded as offsetting or compensating for each other.

ORIGIN OF LUNATICS UNDER TREATMENT IN 1853.

Among 30,084 cases whose origin has been ascertained, are reckoned 709 foreigners, or 23 to 1000. Of these there were:—

| | |
|---|---|
| Males . . . . . . . . . . . . . . | 421, or 25 to 1000 |
| Females . . . . . . . . . . . . . | 288, or 18 to 1000 |
| Total. . . . . . . . | 709 |

Thus the proportion of men among the foreign lunatics exceeds that of the women by more than one-third. An easy explanation of this difference is the fact that the men are the greater travellers, and of course more liable to be found away from home.

The single department where are found the greatest number of foreign lunatics, is that of the Seine. Of 1234 patients under treatment in this department in 1853, 187 were of foreign origin, which gives a proportion of 36 per cent. To explain this predominance we have only to remember that the department contains Paris, a city in whose population we should naturally look for a considerable foreign element.

A *resumé* of the facts under this head furnished by the Report, shows that mental affections are much more frequent among individuals born in those localities in the vicinity of the Seine, and also those which lie in the north-west of France—i.e., Bretagne and Normandy, while insanity is much less common among individuals born in the southern departments and the mountainous districts. These observations remain to be confirmed by further investigations on the part of specialists.

In examining the economical condition of those departments having the largest or the least number of lunatics born within their respective limits, we observe that four of the departments most remarkable for their industrial interests, stand at the head of those furnishing the greatest number of cases. These are, Bouches-du-Rhône, Seine, Seine-Inférieure, and Rhône.

